# Fruits All the Year Round

**Published:** 1866-07

**Authors:** 


Fruits All the Year Round.—We hope the time is not far distant when every three or four farmers adjoining each other will build fruit houses which will preserve berries and grapes and fruits for another year, and at the end of that time be as hard, crisp and not fully ripened up as when just put in. The comfort, the luxury and the advantage of this, to say nothing of the increased healthfulness which would be insured to any family which would consume fruits, berries and grapes largely every day in the year, would be incalculable. Professor Bryce has patented a structure for this purpose. in full operation at Trenton, New Jersey. Samuel H. Rebbins, of Bristol, Penn., is agent for the Patentee. The structure of a large house costs about one dollar for every bushel it holds. A house with a room 15 feet square and eight feet high, being 23 feet square on the outside, holding 500 bushels, would cost about eight hundred dollars. We are not acquainted with the parties, but give this notice for the benefit of our readers as a public good ; the principles involved are the maintenance of a dry temperature just above the freezing point, or thirty-four degrees, by means of a small expenditure of ice, and the absorption of the dampness in the atmosphere.

